# Metabolomics study of fibroblasts damaged by UVB and BaP

**DOI:** 10.1038/s41598-021-90186-7

**Published:** 2021-05-27

**Authors:** Xiaoyu Yang, Jiateng Wang, Hecong Wang, Xueying Li, Congfen He, Lei Liu

**Affiliations:** Beijing Key Laboratory of Plant Resources Research and Development, College of Chemistry and Meterials Engineering, Beijing Technology and Business University, Beijing, 100048 China

**Keywords:** Biotechnology, Cell biology

## Abstract

We have recently shown that both UVB and BaP can induce the production of ROS, apoptosis and even cancer. However, the differences in the metabolic profiles of skin damaged by UVB, BaP or UVB combined with BaP have not been studied. Therefore, we examined the metabolic changes in the human foreskin fibroblast injured by UVB or BaP or the combination of the two, using ultra performance liquid chromatography (UPLC) coupled with quadrupole time-of-flight mass spectrometry (qTOF-MS). 24 metabolites were altered in the UVB damage group, 25 in the BaP damage group, and 33 in the UVB combined with BaP group. These alterations indicated that the metabolic mechanisms of HFF-1 cells treated with UVB or BaP are related to multiple main metabolites including glycerophosphocholine (PC), lactosylceramide (LacCer), guanidinosuccinic acid (GSA), glutathione(GSH), and lysophosphatidylcholine (LysoPC) and the main mechanisms involved glycerophospholipid and glutathione metabolism. Thus, our report provided useful insight into the underlying mechanisms of UVB and BaP damage to skin cells.

## Introduction

Ultraviolet B (UVB) is one of the exogenous aging factors, which causes skin senescence through the accumulation of external stimuli^1^. In the IARC report, 91% of the sunlight on the earth's surface is visible light, with wavelengths ranging from 400 to 800 nm; 8.7% for UVA, 320 to 400 nm; 0.3% for UVB, and too short to 280–320 nm, UVC, all are absorbed by the ozone layer^2,3^. Different depths of penetration of UVA and UVB into the skin will have different effects, inducing different overlapping biological responses in the epidermis and dermis^4^.

When fibroblasts are irradiated with UVB, the biomolecules of the cells can be activated by an endogenous photosensitiser to an excited state and produce singlet oxygen (O_2_^·−^), ^·^H_2_O_2_ and active nitrogen radicals such as NO^5,6^. Significant decreases in triglycerides and total free fatty acids have been found in the epidermis after acute and chronic exposure of human skin to UV radiation^7^, and these changes in free fatty acids in the skin after UV treatment have been associated with fatty acid synthesis^8^. Fatty acids played an important role in controlling inflammation in UVB-irradiated mouse skin tests, especially saturated fatty acids^8^.

Polycyclic aromatic hydrocarbons (PAHs) make up a large class of toxic organic pollutants. Low levels of PAHs are ubiquitous in the environment, found in air, water, and soil, and long-term contact with the skin increases risks of cancer^9,10^. Benzo[a]pyrene (BaP) was the first PAHs to be discovered and is representative of PAHs with its chemical stability, wide distribution and correlations with other PAHs. While nontoxic itself, in that it cannot directly damage covalently linked proteins and DNA, after being metabolised it can be directly absorbed by the lungs or skin and cause damage^11,12^. When BaP is irradiated by visible light (400–700 nm) and UV regions (290–400 nm), it can be activated to a photo-excited state and transform into derivatives like oxygenated BaP and BaP quinones^13^. These metabolites can react with DNA to form covalently bound DNA adducts, induce cytokine, induce ROS production and/or induce apoptosis, even acting through a series of changes to increase the chances of carcinogenesis^11,14,15^. In today's environment, our skin is damaged by both ultraviolet rays and pollutants at the same time. However, research on the synergistic effects of ultraviolet light and pollutants is rare, and the commonly used research methods may not have been comprehensive. Therefore, we used cell metabolomics to study the mechanisms by which UVB and BaP synergistically injure skin cells.

In this study, we investigated the toxicological effects of acute UVB photodamage and short-term low-dose BaP exposure on the overall metabolic profile of human cells. Metabolites were identified by liquid chromatography/mass spectrometry (UPLC/MS)^38^. Using the data produced, potentially important metabolic processes and pathways were explored using bioinformatics analysis. This study characterised metabolism after chronic and environmental BaP exposure to human fibroblasts (HFF-1), and hence, contributed to a more comprehensive understanding of the toxicity of UVB and BaP.

## Materials and methods

### Cell cultures

HFF-1 cells were provided by the Stem Cell Bank, at the Chinese Academy of Sciences. Cells were maintained in high glucose Dulbecco’s Modified Eagle Medium (DMEM) with 10% foetal bovine serum (PBS) and 1% penicillin–streptomycin solution in a humidified atmosphere of 5% CO_2_ at 37 °C. When sub-cultured to about 90% confluence, the HFF-1 cells were seeded into culture flasks or well plates.

### UVB exposure and Cell viability assay

Prior to the experiment, Human foreskin fibroblast (HFF-1) cells were seeded at 50,000 cells/mL in 96-well plates incubated for 24 h. The cells were divided into two groups: (1) UVB blank control group: kept in a thin layer of PBS without UVB exposure; (2) UVB groups: cells were placed inside UVB in a thin layer of PBS and serially irradiated in doses of 5, 10, 15, 20 mJ/cm^2^. To evaluate the toxicity after acute UVB irradiation and determine the appropriate dose of UVB, cell viability was determined using the CCK-8 kit and measuring optical density at 450 nm.

### BaP exposure and ROS assay

HFF-1 cells were divided into the blank control group, negative control group (0.1% DMSO), positive control group and BaP damage group to select the concentration of BaP for further use. The cells were grown in 24-well plates for 24 h, followed by exposure to the predetermined doses of UVB. Then the BaP damage group was treated with culture media supplemented with 10 nM to 100 μM BaP, the negative control group was treated with 0.1% DMSO/culture media, and the other groups were only incubated with culture media.

After 24 h BaP treatment, intracellular ROS generation of HFF-1 cells were measured using the oxidation-sensitive dye 2′,7′-dichlorofluorescin diacetate (DCFH-DA). All groups were then incubated for 20 min with DCFH-DA. All cells were centrifuged and rinsed twice with phenol red-free DMEM (PRD) to clear the DCFH-DA. Cells were detected at an excitation wavelength of 485 nm and an emission wavelength of 535 nm using a microplate reader for ROS detection.

### Sample preparation

HFF-1 cells were divided into four groups: control group (C), UVB exposure group (U), BaP exposure group (B), UVB and BaP group (D). C and D were exposed to UVB in a thin layer of PBS which was then replaced with the fresh culture medium, and in groups B and D, 10 μM BaP was then added. Afterward, cells were incubated for another 24 h before the culture medium was discarded and cells were washed twice at 37 °C with PBS in order to reduce temperature shock to cells. Cells from each group were flash-frozen in liquid nitrogen and immediately stored in a − 80 °C freezer 5until further analysis.

Prior to metabolomics analysis, taking out the cell samples in a − 80 °C freezer, and briefly thawed on ice ,then vortexed with 1 mL cold methanol/chloroform/H_2_O (8:1:1, v/v/v) for 1 min. After that scraped off the cells with a cell scraper, Cells of each sample were detached and collected into a centrifugation tube and sonicated at 4 °C for about 10 min. Cells lysates were then incubated for 30 min on ice and centrifuged at 4 °C for 10 min, whereupon the supernatant was dried under a nitrogen stream. Residues were re-suspended in 200 μL acetonitrile/H_2_O (1:1) and centrifuged again. The upper layer was then transferred to a deactivated glass vial and stored at 4 °C for UPLC-QTOF-MS analysis. The QC samples were inserted 6 samples at a time throughout the run to ensure the stability of the instrument.

### UHPLC-QTOF-MS analysis

All samples were analysed with an UPLC-QTOF-MS system. Briefly, full scan spectra between 50 and 1200 Da were acquired, and injected into an Acquity UPLC BEH C18 column (2.1 mm × 100 mm, 1.7 μm, Waters, USA). The injection volume was 5 μL and the column temperature was maintained at 50 °C. The phases were (A) water with 0.1% formic acid and (B) acetonitrile with 0.1 formic acid. The UPLC separations were 20 min/sample at a flow rate of 0.40 mL/min using the following scheme: 5% B, increased linearly to 38% B at 10 min, then to 100% B at 15 min, held for 3 min, and decreased linearly to 5% B at 19 min, and held for 1 min.

The mass spectrum was used in the positive and negative electrospray ionisation mode. In both the positive and negative mode, capillary voltage was set at 3200 (+)/2500 (−) V and the sampling cone voltage was 35 V. Nitrogen was used as desolvation gas at a flow rate of 800 L/h at 400 °C. The ionisation source temperature was set at 120 °C. Data were collected in centroid mode. Samples were run in a random sequence.

### Data processing

Metabolomics data were processed using Markerlynx XS and EZ info software (Waters) combined with the Human Metabolome Database (HMDB). The (O)PLS-DA or PLS-DA methods were used to further analyse the data provided by the HMDB. Then, biomarker identification was conducted according to variable importance in projection values (VIP > 1), using an independent t-test (*P* < 0.05), accurate molecular ions were obtained by MS (mass error < 5 ppm), and fragment ions were obtained by MS/MS. The respective metabolic pathways of marker metabolites were mapped using Metaboanalyst 4.0 (https://www.metaboanalyst.ca/).

Statistical significance was calculated by Student’s t-test or analysis of variance (ANOVA) using IBM SPSS Statistics 21.0.0.0. False discovery rate (FDR) correction was calculated to reduce the risk of a false-positive by the adjusted p values (< 0.05) based on Holm-Bonferroni method. For all analyses, ***P < 0.001; **P < 0.01; *P < 0.05.

## Results

### Cytotoxicity after UV irradiation

**Figure 1:**
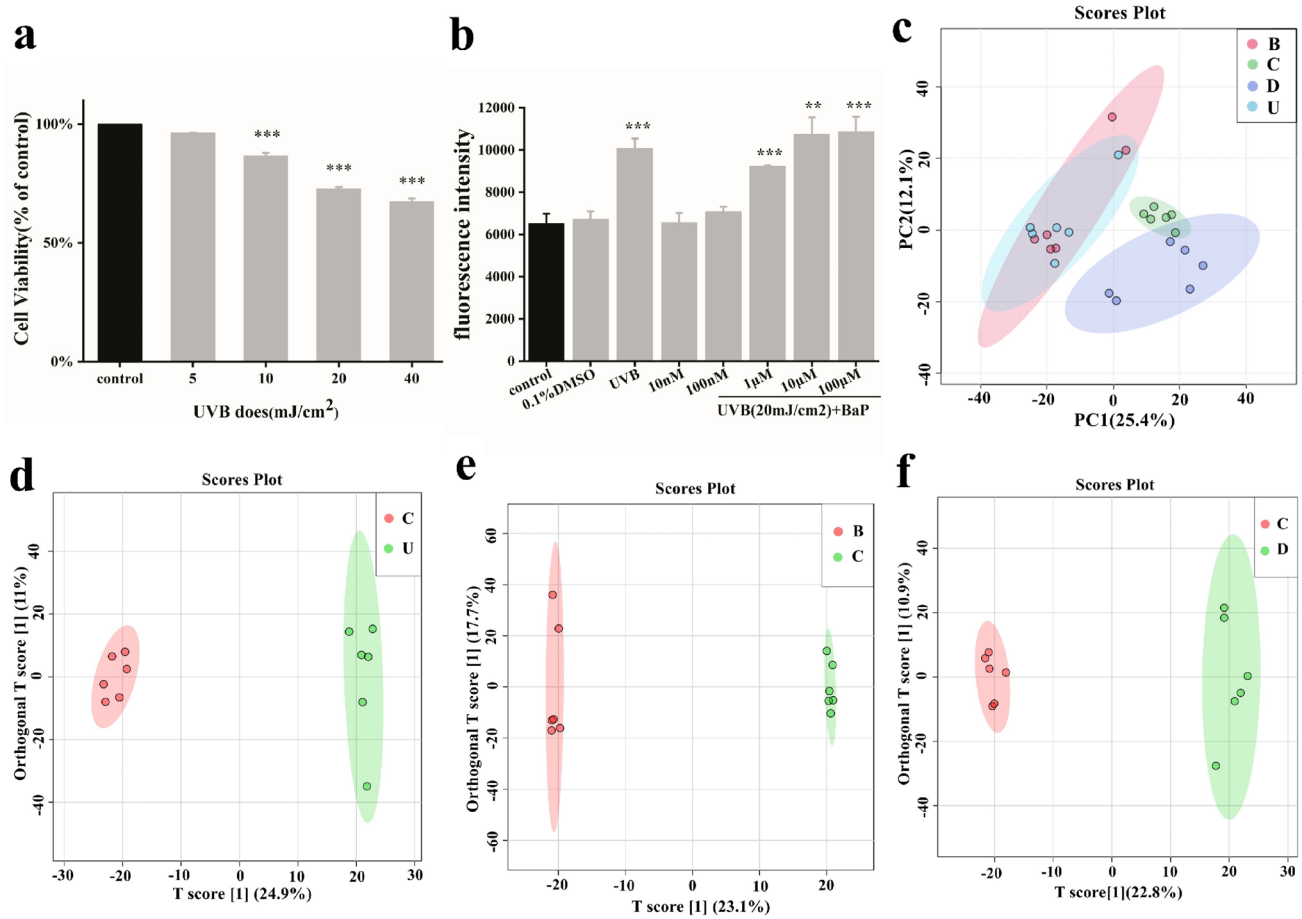
Principal components analysis for metabonomics of damaged cell models. (**a**) Cytotoxicity of HFF-1 exposed to different UVB irradiation intensities. (**b**) BaP induced reactive oxygen species production. Cells were exposed to 5–40 mJ/cm^2^ UVB irradiation and the doses of BaP ranged from 10 nM to 100 µM. (**c**) PCA score plots. (**d**–**f**) OPLS-DA score plots based on the data from UPLC-ESI (+)-QTOF-MS for distinguishing C versus U (R2Y = 99.6%, Q2 = 85.6%), C versus B (R2Y = 99.6%, Q2 = 86.1%), and C versus D (R2Y = 99.4%, Q2 = 86.9%) for selecting potential markers.

To determine the cytotoxicity of UVB irradiation, cells were irradiated by UVB ranging from 5 to 40 mJ/cm^2^ after incubation for 24 h (Fig. 1a). The survival rates of HFF-1 cells were 93%, 81%, 70% and 65%, respectively. When the radiation dose of UVB reached 40 mJ/cm^2^, the cell viability showed obvious concentration gradient dependence (p < 0.01). It is generally believed that the 70% survival rate indicates that the injury effect can be determined, and the dose of 20 mJ/cm^2^ is selected for further use.

### Intracellular ROS generation

BaP (10 NM- 100 μ M) treatment had no effect on the viability of HFF-1 cells in the short term, but UVB and BaP could affect the ROS content of cells^3,4,11,14^, so we chose ROS as the modeling parameter. Followed irradiation by UVB with BaP (10 nM–100 μM) didn’t affect the viability of HFF-1 cells (data not shown). UVB irradiation and BaP both lead to increased oxidation in the human skin cells.Therefore, the cytotoxicity of BaP against HFF-1 cells was determined by measuring the level of intracellular ROS generation. The results showed that ROS levels in HFF-1 cells showed dose-dependent on BaP and reached the highest value at a dose of 10 μM (Fig. 1b). Thus, 10 µM BaP was selected for further use.

### Multivariate statistical analysis

The preprocessed data were subjected to multivariate analysis for sample classification. PCA-class analysis was first performed to evaluate the similarity of the samples within each class (Fig. 1c). To further investigate changes in metabolites among the C, B, D and U groups, we applied the OPLS-DA method to identify differences between groups, as shown in the figure (Fig. 1d–f). The OPLS-DA was further validated using permutation test, the regression line of the Y-permuted Q2 was intercepted at − 0.172 (C versus B), − 0.379 (C versus U) and − 0.0284 (C versus D) respectively, indicating a reliable predictive power of the model.

**Figure 2:**
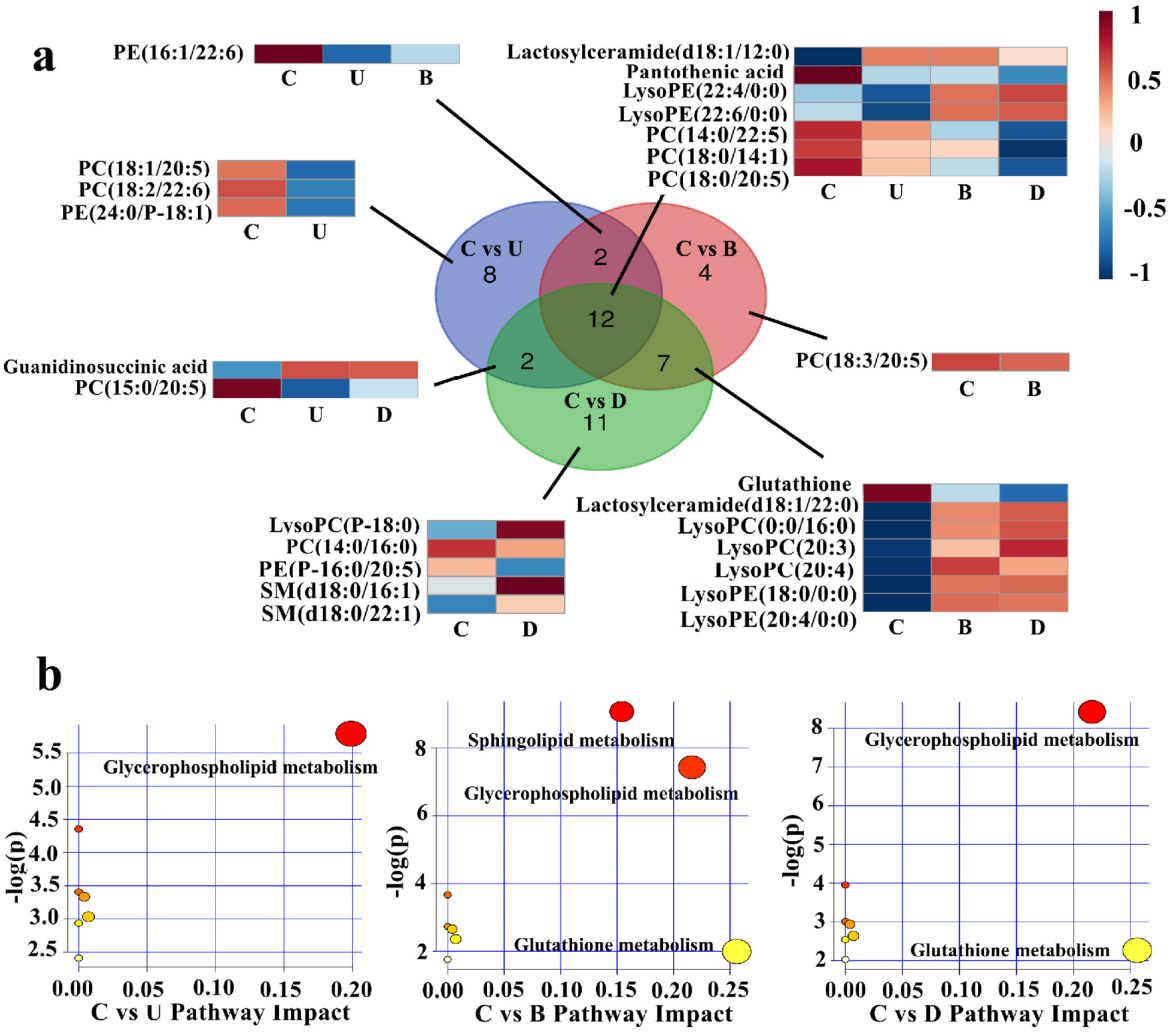
Differential metabolites of damaged cell models. (**a**) Overlap analysis of metabolites differing between damaged groups and the control group. (**b**) Pathway analysis showing altered metabolic pathways. C, control group; U, UVB exposure group; B, BaP exposure group; D, UVB and BaP group.

Subsequent processing with VIP analysis, VIP value larger than 1.0 were selected as putative biomarkers and are summarised in Fig. 2. After statistical analysis, Zero or missing values were replaced by 1/5 of the min positive value for each variable, and no data filtering was performed. C versus U and C versus B were found to have 18 altered metabolites in common, 12 of which also appeared in C versus D. Then we use the ANOVA and find that 11 substances have P ≤ 0.05, except for Pantothenic acid (vip > 1, p = 0.0655), so the remaining 11 substances except Pantothenic acid can be used as biomarkers. And there is no missing value in these biomarkers.

Metabolomic pathway analysis was used to examine signal pathway enrichment analysis in the MetPA (Metabolomic Pathway Analysis) software. A total of 7 metabolic pathways were disrupted in all three experimental groups, which represented all three types of damage to HFF-1 cells, with glycerophospholipid metabolism changing most significantly, and glutathione metabolism changing only in C versus B and C versus D. The summary of the pathway analysis is shown in Fig. 2 with the detailed results of pathway analysis in Table 1. High glucose cultures may cause cell senescence, but this experiment set up a control group to eliminate the effect, and will not affect the experimental results.

**Table 1:** Pathway analysis of differential metabolites of damaged cell models.

	Total	Expected	Hits	Raw p	Holm adjust	FDR	Impact	Pathway group
Glycerophospholipid metabolism	36	0.093	2	0.0030	0.257	0.257	0.199	C versus U
Linoleic acid metabolism	5	0.013	1	0.0128	1	0.540	0	
alpha-Linolenic acid metabolism	13	0.033	1	0.0331	1	0.744	0	
Glycosylphosphatidylinositol (GPI)-anchor biosynthesis	14	0.036	1	0.0356	1	0.744	0.004	
Pantothenate and CoA biosynthesis	19	0.050	1	0.0482	1	0.744	0.007	
Sphingolipid metabolism	21	0.054	1	0.0531	1	0.744	0	
Arachidonic acid metabolism	36	0.093	1	0.0898	1	1	0	
Sphingolipid metabolism	21	0.108	3	0.0001	0.010	0.010	0.154	C versus B
Glycerophospholipid metabolism	36	0.186	3	0.0006	0.049	0.025	0.216	
Linoleic acid metabolism	5	0.026	1	0.0256	1	0.716	0	
alpha-Linolenic acid metabolism	13	0.067	1	0.0653	1	1	0	
Glycosylphosphatidylinositol (GPI)-anchor biosynthesis	14	0.072	1	0.0702	1	1	0.004	
Pantothenate and CoA biosynthesis	19	0.098	1	0.0941	1	1	0.007	
Glutathione metabolism	28	0.144	1	0.1360	1	1	0.256	
Arachidonic acid metabolism	36	0.186	1	0.1717	1	1	0	
Glycerophospholipid metabolism	36	0.139	3	0.0002	0.018	0.018	0.216	C versus D
Linoleic acid metabolism	5	0.019	1	0.0192	1	0.808	0	
alpha-Linolenic acid metabolism	13	0.050	1	0.0494	1	1	0	
Glycosylphosphatidylinositol (GPI)-anchor biosynthesis	14	0.054	1	0.0531	1	1	0.004	
Pantothenate and CoA biosynthesis	19	0.074	1	0.0714	1	1	0.007	
Sphingolipid metabolism	21	0.081	1	0.0787	1	1	0	
Glutathione metabolism	28	0.108	1	0.1038	1	1	0.256	
Arachidonic acid metabolism	36	0.139	1	0.1317	1	1	0	

## Discussion

### Overlap metabolites of UVB and BaP damage to HFF-1

Among the metabolites that responded to UVB damage, BaP damage, and the combination of the two in HFF-1 cells, there were 12 metabolites that responded in all three cases. Among them, glycerophosphocholine (PC), one of the main components of cell membranes, had the most significant change. The relative content showed PC was most abundant in the control group (group C), and had decreased in the three treatment groups. After HFF-1 treatment with UVB or BaP, the contents of either PC or glycerophosphoethanolamine (PE) were reduced due to the peroxidation of phospholipids by increased ROS. Therefore, the increasing permeability of the cell membrane became increasingly susceptible to external stimuli^16^, and small molecules such as BaP could pass through more easily. Lysophosphatidylcholine (LysoPC) and lysophosphatidylethanolamine (LysoPE) were the intermediate products of the PC and PE oxidation processes, respectively^17,18^. In addition, the relative contents of LysoPE (22:4/0:0) and LysoPE (22:6/0:0) were greater in the two groups containing BaP damage than the UVB only group. Combined with what we can see in Fig. 1b, BaP played a larger role than UVB in oxidation.

Lactosylceramide (LacCer) activates an "oxygen-sensitive" signalling pathway involving superoxides, nitric oxide, kinase cascade and nuclear factor up-regulation, eventually causing cancer and inflammatory diseases^19^. In general, the major source of LacCer was from the conversion of glucosylceramide and GM3, which raises the level of ROS and activates phosphorylation of mitogen-activated protein kinases^20^. After exposure to UVB and BaP, increasing levels of LacCer activated phospholipase A-2 in neutrophils, thus releasing arachidonic acid and eicosanoids, and causing skin inflammation^21^.

### Differences in HFF-1 metabolites after UVB and BaP damage

**Figure 3:**
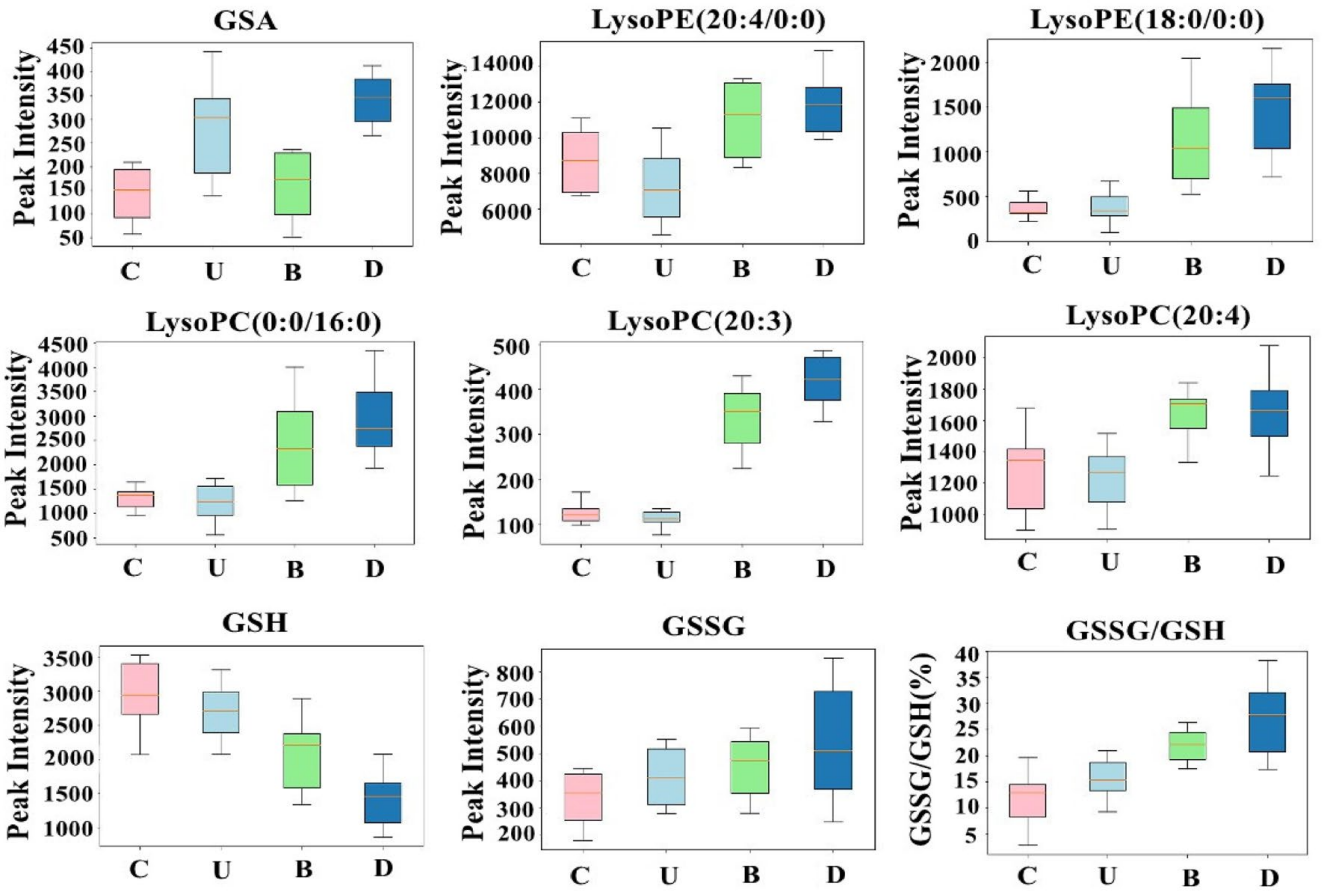
Boxplot of the altered metabolites. GSA was main biomarker in C versus U and C versus D. LysoPE, LysoPC and GSH were the main biomarkers in C versus B and C versus D. GSSG and the ratio of GSSG/GSH represented oxidative stress of cells, the larger the ratio of GSSG/GSH, the stronger the oxidation. C, control group; U, UVB exposure group; B, BaP exposure group; D, UVB and BaP group.

In groups U and D, guanidinosuccinic acid (GSA) content was higher than group C (as shown in Fig. 3). GSA, a stable NO mimic, was generated from argininosuccinate (ASA) and the hydroxyl radical synthesised from a combination of NO and a superoxide anion^22^. This process was accompanied by the production of gamma glutamic semialdehyde, an advanced glycation end product, which commonly indicates the Maillard reaction has occurred^23^. The Maillard reaction is one of the common reactions of skin ageing due to collagen cross-linking.

Among the metabolites in common between groups B and D, glutathione (GSH), LysoPC and LysoPE were identified as biomarkers. As shown in Fig. 3, the increased levels of LysoPC and LysoPE and the decreased levels of GSH reflected the higher degree of oxidation in groups B and D compared to group U. GSH was could be used as a representative biomarker for the oxidation process. The decreasing GSH content, and increasing glutathione disulphide (GSSG) and GSSG to GSH ratio shown in Fig. 3 are similar to the findings of most previous cell oxidation experiments. A rapid enzymatic process that utilises, degrades, or regenerates GSH will stabilise the ratio of GSSG to GSH to reflect a steady state^24^. The ratio of GSSG to GSH was used as an effective tool to study redox metabolism. It was reported that the GSSG to GSH ratio and GSSG were increased, and that GSH was reduced by oxidative damage in cells exposed to UVB or BaP^25–27^. During oxidative stress, two GSH molecules tends to be linked by a disulphide bridge to form GSSG^28^, thus the oxidant-antioxidant balance was tipped in favour of the former. The ratio of GSH/GSSG has been seen to decrease to about 10:1, or even 1:1^29^. As for its role, GSH detoxified ROS and regenerated oxidised α-tocopherol and retinol as anti-oxidants, while it decreased hydrogen peroxide to water and protected lipids from peroxidation^30^.

Dysregulated LysoPC, which is supposed to play an important role in introducing and maintaining the inflammatory response, may constitute an important part of the disease development and progression^31^. LysoPC acts as the main inducer of complex physiological responses in different kinds of signals and cells. For example, LysoPC induced the blood brain barrier in rat brain in addition to an immunological response^32^. In keratinocytes, LysoPC was able to induce differentiation due to protein kinase C by increasing the expression of transglutaminase-1^33^. It was also reported that LysoPC has the potential of cytoprotective and anti-inflammatory activity^34^. LysoPC may affect the degree of oxidation of cells by affecting the lipid composition of the mitochondrial membrane^17,18^. The higher the oxidation intensity of PC in cells, the more highly unsaturated PC will be oxidised^35^. The additive effects of BaP and UVB exacerbated the oxidative stress response of HFF-1 cells, and increased LysoPC may have enhanced the inflammatory effect or may have protected damaged cells.

### Pathway analysis

The glycerophospholipid metabolism and glutathione metabolism pathways were very important metabolic pathways for HFF-1 cells exposed to UVB and BaP. They were also found to be important in psoriasis^36^and in HaCaT cells exposed to phenanthrene^37^. According to the ROS level test, the ROS level of the combined UVB and BaP injury group was high, and the relative contents of PC, PE, LysoPC and GSH were changed. This result means that ROS-induced oxidative damage by UVB and BaP in HFF-1 cells was closely related to the role of the glycerophospholipid metabolism pathway. As for glutathione metabolism, the change in GSH content (Fig. 3) suggested that it was affected by UVB, but especially affected by BaP and BaP combined with UVB.

## Conclusion

In this test, metabolomics analysis was used to study the damage to HFF-1 cells caused by UVB and BaP. The injured (experimental) groups and the control group were significantly different and showed significant metabolite disturbance. With the assistance of the high resolution and high accuracy MS/MS data, biomarkers, including PC, LysoPC, LacCer, GSH and other specific metabolites, were identified. The glycerophospholipid metabolism and glutathione metabolism pathways emerged as the key pathways involved in the dysregulation by the oxidative damage. This study has provided new insight into the underlying mechanisms of UVB and BaP damage to skin cells, and has demonstrated that cell metabolomics can provide clear insight into the mechanisms of combined injury.

## Abbreviations

ASA: Argininosuccinate
BaP: Benzo[a]pyrene
CoA: Coenzyme A
DCFH-DA: Oxidation-sensitive dye 2′,7′-dichlorofluorescin diacetate
DMEM: High glucose Dulbecco’s Modified Eagle Medium
DPE: Diesel particulate extract
EGF: Epidermal growth factor
EGFR: Epidermal growth factor recepter
GM3: NeuAcα2 → 3Galβ1 → 4Glcβ1 → 1Cer.
GSA: Guanidinosuccinic acid
GSH: Glutathione
GSSG: Glutathione disulphide
HaCaT: Human Keratinocytes Cell
HFF-1: Human fibroblasts cells
HMDB: Human Metabolome Database
IL: Interleukin
LacCer: Lactosylceramide
LysoPC: Lysophosphatidylcholine
LysoPE: Lysophosphatidylethanolamine
PA: Phosphatidic acid
PAHs: Polycyclic aromatic hydrocarbons
PBS: Phosphate buffered saline
PC: Glycerophosphocholine
PE: Glycerophosphoethanolamine
PG: Phosphatidylglycerol
PS: Phosphatidylserine
SM: Sphingomyelin
TG: Monodocosahexaenoic acid triglyceride
TNFα: Serum tumor necrosis factor
